# Employing phylogenetic tree shape statistics to resolve the underlying host population structure

**DOI:** 10.1186/s12859-021-04465-1

**Published:** 2021-11-10

**Authors:** Hassan W. Kayondo, Alfred Ssekagiri, Grace Nabakooza, Nicholas Bbosa, Deogratius Ssemwanga, Pontiano Kaleebu, Samuel Mwalili, John M. Mango, Andrew J. Leigh Brown, Roberto A. Saenz, Ronald Galiwango, John M. Kitayimbwa

**Affiliations:** 1 Institute of Basic Sciences, Technology and Innovation (PAUSTI), Pan African University, Nairobi, Kenya; 2 Department of Mathematics, Makerere University, Kampala, Uganda; 3 Uganda Virus Research Institute (UVRI), Entebbe, Uganda; 4 Department of Immunology and Molecular Biology, Makerere University, Kampala, Uganda; 5 UVRI Centre of Excellence in Infection and Immunity Research and Training (MUII-Plus), Makerere University, Entebbe, Uganda; 6 Centre for Computational Biology, Uganda Christian University, Mukono, Uganda; 7 Medical Research Council (MRC)/Uganda Virus Research Institute (UVRI) and London School of Hygiene and Tropical Medicine (LSHTM) Uganda Research Unit, Entebbe, Uganda; 8 Department of Statistics and Actuarial Sciences, Jomo Kenyatta University of Agriculture and Technology, Nairobi, Kenya; 9 Institute of Evolutionary Biology, University of Edinburg, Edinburg, UK; 10 Facultad de Ciencias, Universidad de Colima, Colima, Mexico

**Keywords:** Structured, non-structured, Host population, Phylogenetic tree, Simulation, Tree statistics, Classification

## Abstract

**Background:**

Host population structure is a key determinant of pathogen and infectious disease transmission patterns. Pathogen phylogenetic trees are useful tools to reveal the population structure underlying an epidemic. Determining whether a population is structured or not is useful in informing the type of phylogenetic methods to be used in a given study. We employ tree statistics derived from phylogenetic trees and machine learning classification techniques to reveal an underlying population structure.

**Results:**

In this paper, we simulate phylogenetic trees from both structured and non-structured host populations. We compute eight statistics for the simulated trees, which are: the number of cherries; Sackin, Colless and total cophenetic indices; ladder length; maximum depth; maximum width, and width-to-depth ratio. Based on the estimated tree statistics, we classify the simulated trees as from either a non-structured or a structured population using the decision tree (DT), K-nearest neighbor (KNN) and support vector machine (SVM). We incorporate the basic reproductive number ($R_0$) in our tree simulation procedure. Sensitivity analysis is done to investigate whether the classifiers are robust to different choice of model parameters and to size of trees. Cross-validated results for area under the curve (AUC) for receiver operating characteristic (ROC) curves yield mean values of over 0.9 for most of the classification models.

**Conclusions:**

Our classification procedure distinguishes well between trees from structured and non-structured populations using the classifiers, the two-sample Kolmogorov-Smirnov, Cucconi and Podgor-Gastwirth tests and the box plots. SVM models were more robust to changes in model parameters and tree size compared to KNN and DT classifiers. Our classification procedure was applied to real -world data and the structured population was revealed with high accuracy of $92.3\%$ using SVM-polynomial classifier.

## Background

A number of evolutionary, demographic, environmental, epidemiological and immunological factors greatly impact on the genetic variation in a given population [1, 2]. These genetic variations can be summarised as phylogenetic trees, from which the determining factors can be empirically estimated [2, 3]. A phylogenetic tree consists of nodes, branches and tips representing a hypothesis of evolutionary relationships among genes, organisms, species and populations from a common ancestor [2, 4]. A phylogenetic tree is fully described by its tree topology (e.g. branching patterns) and branch lengths [2, 5–7]. The tree topology is described by the branching patterns arising from events such as birth, death, migration and sampling among the populations being analysed [6]. Birth and death correspond to speciation and extinction of species, respectively [6, 8]. Sampling allows species or infected hosts to be included into the phylogeny [9, 10]. The techniques that are employed to model the branching patterns in the phylogeny are coalescent and birth-death processes [11–14]. A phylogeny can be constructed by simulating the branching patterns or by using simulated sequences or genomes [15, 16]. The underlying structure of the host population can be determined from a tree that is reconstructed using genomes from randomly sampled individuals coupled with their demographic characteristics [17]. This is usually done by analysing the clustering and balance of taxa on the resultant tree.

A structured population is characterised by types (sub-populations) or demes [3, 11, 17]. In epidemics, a population may be structured based on host characteristics such as differences in age, duration of infection, contact rate, infectiousness and susceptibility [18]. The dynamics of an infection that progresses from acute to chronic, and also for a particular disease that infects individuals in separate locations can be modelled using methods for a structured population [11]. Determining how the host population is stratified is essential in capturing the heterogeneity and determining the host characteristics that drive the disease transmission dynamics within and between populations. The model parameters and hypothesis for a structured population with two sub-populations can be tested using maximum likelihood inference [3]. General multi-type birth-death models that extend likelihood inference from two states to multi-states are employed for a structured population with *n* states to estimate epidemiological parameters [17]. In such inference, the contribution of a particular sub-group on the general epidemic is quantified.

Previous phylogenetic studies have utilised genomic data for reconstructing time-resolved phylogenetic trees to study the evolution and transmission trends of pathogens and infections in populations [17, 19–21]. This has been achieved by analysing the inferred tree shape, which is one of the most important properties of a phylogeny [22, 23]. The tree’s shape describes the tree’s topology or branching pattern and branch lengths. The tree’s topological features are referred to as the tree’s statistics [24]. The topological features of a tree can be analysed to infer attributes such as the evolutionary process, the dynamics (e.g. the basic reproductive number, $R_0$) and patterns of transmission of an epidemic [25]. Furthermore, it can be used to infer important features of a population, such as population size, fitness, ecology and geographical structure [18]. Details such as pathogen selection and immune escape can also be deduced [26]. Tree shapes were used to show that heterogeneity in Human Immunodeficiency Virus (HIV) arises due to differences in the contact rates between groups and differences due to the infectiousness of individuals over the course of an epidemic [18].

**Fig. 1:**
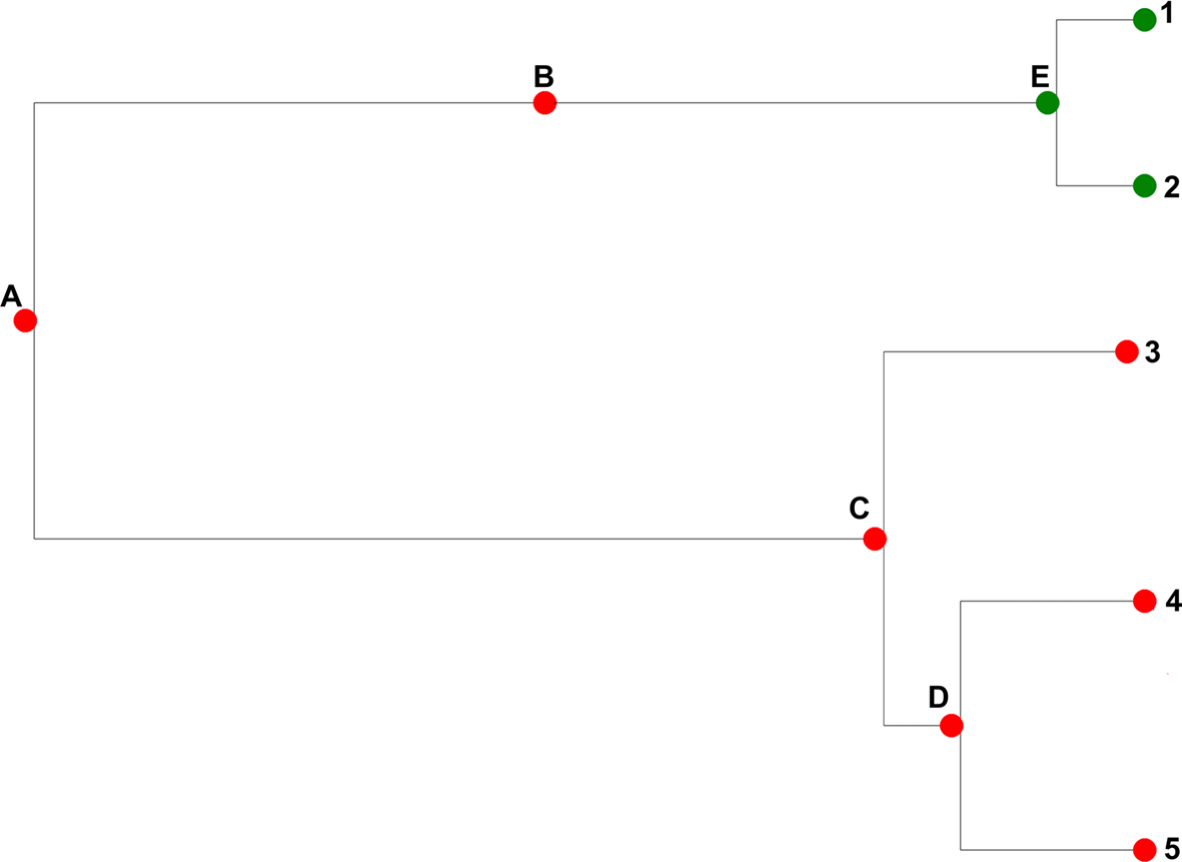
A bifurcating phylogenetic tree simulated using our simulation procedure with 6 tips. Node *A* is the first bifurcation event. Nodes *B*, *C*, *D* and *E* are internal nodes. Nodes numbered 1 up to 6 are the tips or leaves. There is a migration event represented by node *B* to *E*. The phylogenetic tree is from a structured population. The two sub-populations are represented by green and red colours

Tree shapes can be asymmetrical or symmetrical depending on the topological distribution of the taxa among different clades [27]. Tree symmetry is a measure of the degree to which descendants of internal nodes have a similar number of descendant taxa, given as a “balance index” [18, 27, 28]. Topological asymmetry can be assessed both locally and globally [29]. The degree of asymmetry for any given tree can provide support for the hypothesis that species have different potential for speciation [30]. Three main statistics have been used to measure tree symmetry, which are: number of cherries, Sackin and Colless indices [24, 30, 31]. It was showed that the number of cherries is asymptotically normal as the number of taxa grows to infinity under both Yule and uniform models [32]. In other studies, both the number of cherries and the Sackin index were used to investigate tree asymmetry [18, 29]. Currently, more statistics have been extracted from trees such as total cophenetic index as described in [33]. Other tree statistics include ladder, tree width and depth, among others. A combination of more than one of these statistics can be used to improve inferences from phylogenetic trees [25]. Many of previous studies employ tree statistics for bifurcating trees and our simulation procedure also resulted in bifurcating trees. A bifurcating tree with *n* tips has $n-1$ internal nodes. However, by modelling a migration event by a change of colour along a vertical line, the resulting simulated trees with *n* tips have more than $n-1$ internal nodes. These are as well bifurcating since for a migration event, it is the same individual moving from one sub-population to the other (see Fig. 1).

In this study, we simulated birth-death trees from both structured and non-structured host populations. For both populations, we then analysed tree shapes by estimating eight tree statistics, namely: number of cherries; Sackin, Colless and total cophenetic indices; ladder length; maximum depth; maximum width, and width-to-depth ratio. We used all the eight tree statistics to classify the simulated trees as either from non-structured or structured populations. In addition, we incorporated $R_0$ in our tree simulation procedure. We investigated whether the classifier models were robust to changes in both parameter values and tree size.

## Results

### Distributions for tree statistics for non-structured and structured populations

**Fig. 2:**
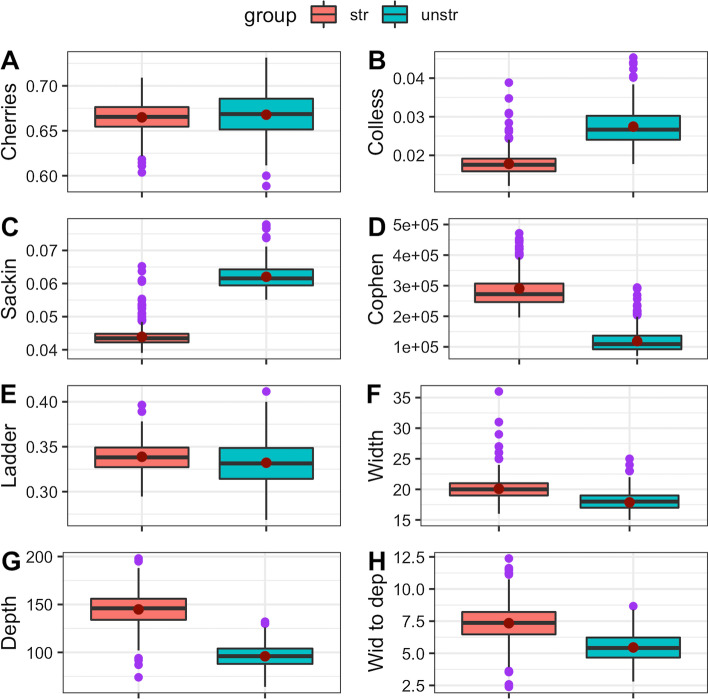
Box plots for tree statistics for dataset 1. The mean values are the red points inside the boxes, the median values are the horizontal black lines inside the boxes and the outliers are the purple dots. The groups were structured (str) and non-structured (unstr). **A** normalised number of cherries, **B** normalised Colles index, **C** normalised Sackin index, **D** total cophenetic index, **E** ladder length, **F** maximum width, **G** maximum depth, **H** width to depth ratio

**Fig. 3:**
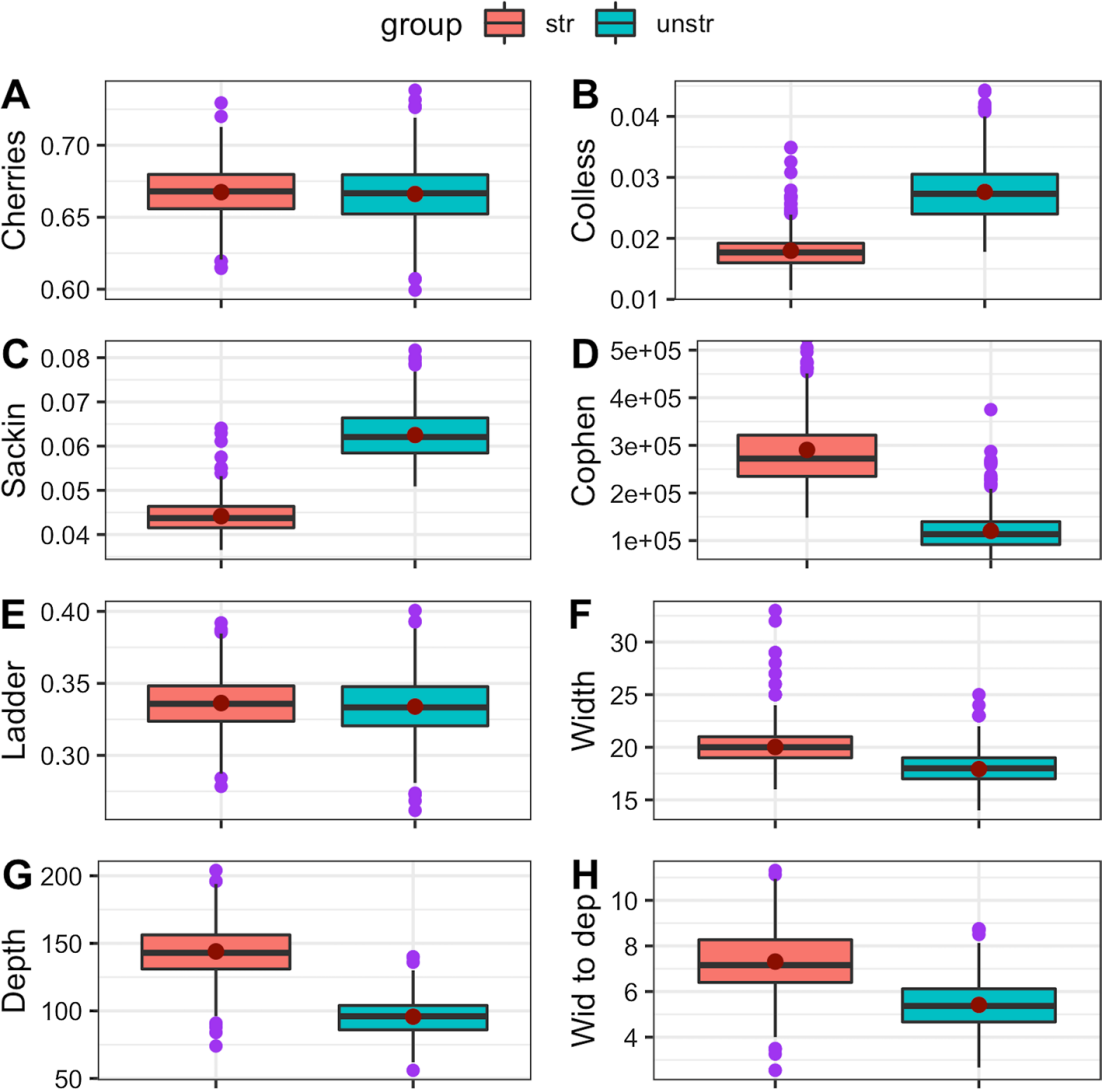
Box plots for tree statistics for dataset 2. The mean values are the red points inside the boxes, the median values are the horizontal black lines inside the boxes and the outliers are the purple dots. The groups were structured (str) and non-structured (unstr). **A** normalised number of cherries, **B** normalised Colles index, **C** normalised Sackin index, **D** total cophenetic index, **E** ladder length, **F** maximum width, **G** maximum depth, **H** width to depth ratio

For the two datasets generated, trees from a non-structured population had higher Colless and Sackin index values compared to trees from a structured population (Fig. 2 & Fig. 3). However, tree index values for cophenetic, maximum depth, maximum depth and width to depth ratio were slightly higher for a structured compared to a non-structured population (Fig. 2 & Fig. 3).

Comparing each tree statistic across the two datasets resulted in some differences. For example, for Colless, Sackin, ladder length, cophenetic and maximum width indices, mean and median values were lower in dataset 1, compared to dataset 2. For the number of cherries and maximum depth, the mean and median values were higher in dataset 1, compared to dataset 2 (Fig. 2 & Fig. 3).

The differences between maximum and minimum (range) values of the tree statistics are slightly higher for trees from non-structured populations compared to trees from structured populations (Fig. 2 & Fig. 3 ). Generally there are some observable differences in distribution and dispersion of tree statistics from structured and non-structured populations.

**Table 1:** Two-sample Kolmogorov-Smirnov, Cucconi and Podgor−Gastwirth tests for comparing distributions of tree statistics between populations for dataset 1. D, C and S are the statistics used in the tests

Tree statistics	Kolmogorov-Smirnov Test		Cucconi Test		Podgor-Gastwirth Test	
	*D*	*p*-value	*C*	*p*-value	*S*	*p*-value
Number of Cherries	0.142	$8.365 \times 10^{-5}$	4.997	0.007	4.951	0.0073
Colless index	0.43	$2.2 \times 10^{-16}$	110.7951	$< 10^{-20}$	142.0913	$<10^{-20}$
Sackin index	0.408	$2.2 \times 10^{-16}$	100.971	$<10^{-20}$	126.3	$<10^{-20}$
Total cophenetic index	0.44	$2.2 \times 10^{-16}$	101.987	$<10^{-20}$	127.896	$<10^{-20}$
Ladder length	0.128	$5.537 \times 10^{-4}$	4.668	0.011	4.567	0.0106
Maximum width	0.362	$2.2 \times 10^{-16}$	62.15007	$<10^{-20}$	79.63062	$<10^{-20}$
Maximum depth	0.304	$2.2 \times 10^{-16}$	50.3996	$<10^{-20}$	56.04892	$<10^{-20}$
Width-depth ratio	0.184	$8.898 \times 10^{-8}$	28.19	$<10^{-20}$	29.825	$<10^{-20}$

Using the two-sample Kolmogorov-Smirnov, Cucconi and Podgor-Gastwirth statistical tests, the difference in structure of a population from which trees were drawn from was identified in all the tree statistics for dataset 1 (Table 1). The difference in structure of a population from which trees were drawn failed to be determined using the number of cherries and the ladder length in dataset 2 (results not shown), though for the rest of the tree statistics, a difference in structure of an underlying population was detected.

### Cross-validated results on tree classification

Optimal parameters were obtained using grid search as implemented in the trainControl function of the “caret” package in R. The optimal parameters include; $k=5$ for KNN; sigma = $0.20$ for SVM-radial; scale = 0.1, degree = $3$ for SVM-polynomial; cp = $0.028$ for DT. The optimal constant *C* of the regularization term in the Lagrange formulation was $C=1$ for all SVM classifiers.

Based on the mean values for the measures (sensitivity, specificity, accuracy and area under the curve (AUC)), the classification performed well for dataset 1 (Baseline dataset; Table 2). Support vector machine (SVM) classifiers performed better than K-nearest neighbor (KNN) and decision tree (DT). For the SVM classifiers, all the mean measures computed were above 0.95 for dataset 1 (Table 2).

**Table 2:** Results of 10-fold cross-validated classification with computed average for the measures for baseline and sensitivity analysis.Times in seconds taken to build respective models are shown as well

Classifier	Sensitivity	Specificity	AUC	Accuracy	Time in seconds
*SVM-linear*					
Baseline	0.99	0.95	0.98	0.97	1.23
Varied tree size	0.98	0.88	0.93	0.93	1.30
Varied parameters	0.93	0.86	0.90	0.90	1.25
Varied tree size & parameters	0.99	0.97	0.99	0.99	1.18
*SVM-polynomia*l					
Baseline	0.99	0.99	0.99	0.99	16.60
Varied tree size	0.98	0.90	0.95	0.94	17.64
Varied parameters	0.99	0.98	0.99	0.99	19.22
Varied tree size & parameters	0.99	0.99	0.99	0.99	16.87
*SVM-radia*l					
Baseline	0.99	0.98	0.99	0.99	2.89
Varied tree size	0.94	0.86	0.94	0.90	2.11
Varied parameters	0.99	0.98	0.99	0.99	2.86
Varied tree size & parameters	0.99	0.98	0.99	0.99	2.27
*Decision Trees*					
Baseline	0.98	0.94	0.96	0.96	1.17
Varied tree size	0.96	0.78	0.85	0.87	1.37
Varied parameters	0.83	0.88	0.84	0.86	1.27
Varied tree size & parameters	0.90	0.85	0.85	0.88	1.29
*KNN*					
Baseline	0.99	0.98	0.99	0.99	1.71
Varied tree size	0.98	0.92	0.97	0.95	1.37
Varied parameters	0.99	0.98	0.99	0.99	1.27
Varied tree size & parameters	0.78	0.84	0.87	0.78	1.29

**Fig. 4:**
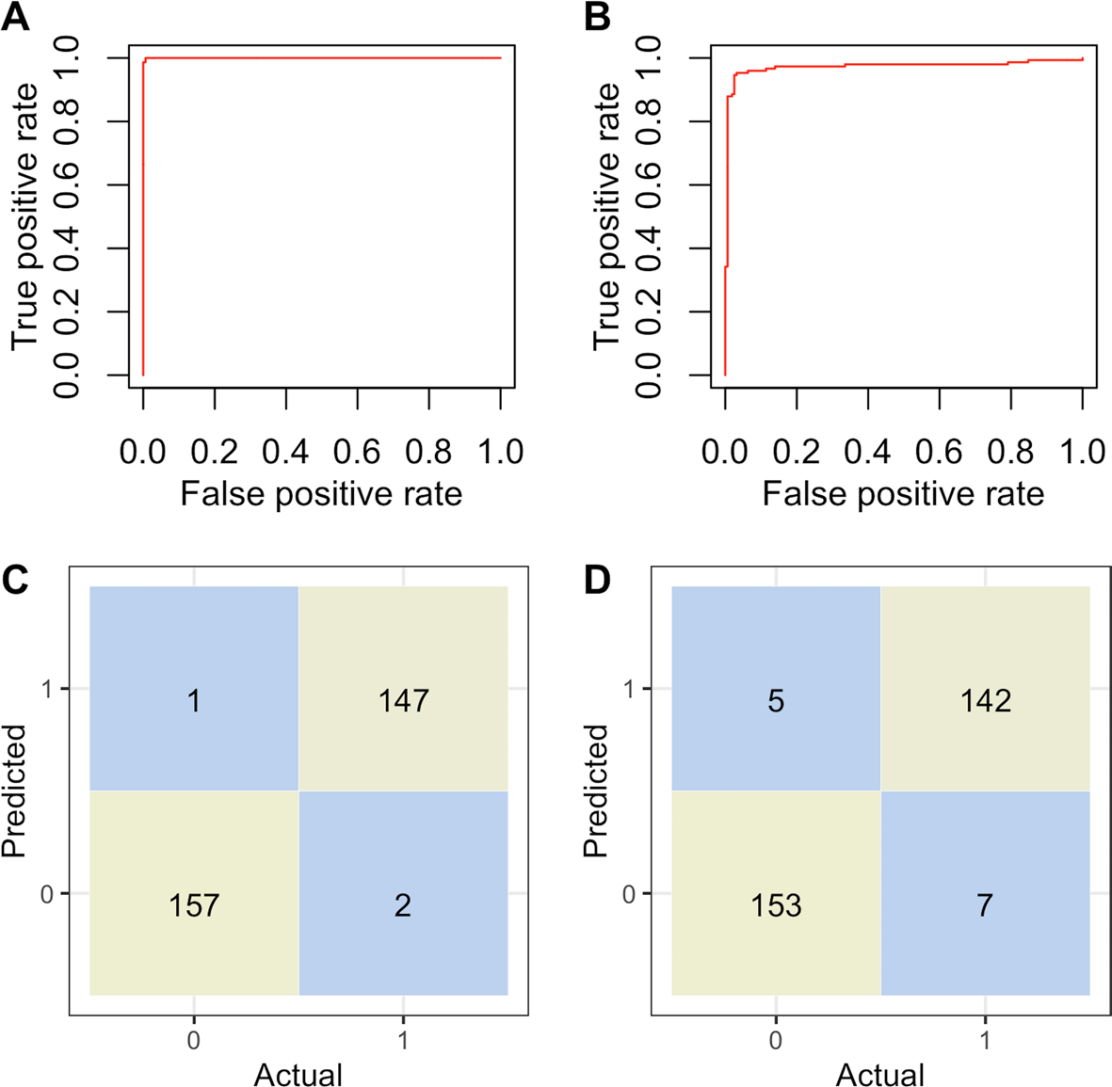
ROC for two of the best classifiers with their corresponding confusion matrices for dataset 1. **A** ROC curve for SVM-radial, **B** ROC curve for SVM-polynomial, **C** confusion matrix for SVM-radial, **D** confusion matrix for SVM-polynomial

Receiver operating characteristic (ROC) curves for SVM-radial and SVM-polynomial, with their corresponding confusion matrices, for dataset 1 are shown in Fig. 4. Both curves indicate that the corresponding area under the curve (AUC) is close to 1. From the confusion matrices shown, few cases ( three out of 307 for SVM-radial and 12 out of 307 for SVM-polynomial) were mis-classified.

### Classification performance on sensitivity analysis

For all SVM models, classifier performance almost remained the same whether tree size, parameters or both tree size and parameters were varied from the baseline (Table 2). In particular for SVM models, varying both tree size and parameters yielded almost no difference in classifier performance when compared to the baseline. However, for KNN and decision trees, differences in performance of classification were registered with baseline performing better than varying tree size and parameters (Table 2). Overall, SVM models were more robust to different choices of model parameters and to the size of the trees compared to decision trees and KNN classification models.

### Performance of the classifiers on real-world data

For general population (GP) and fishing communities (FCs) combined, the SVM-radial classifier most often classified trees as structured $66.3\%$ and $33.7\%$ as non-structured. The SVM-polynomial classified most trees as structured $92.3\%$ and $7.7\%$ as non-structured. In the KNN classification, $64.8\%$ of the trees were classified as structured and $35.2\%$ as non-structured. For trees from the GP only, the SVM-radial classified $66.6\%$ of trees as structured and the $33.4\%$ as non-structured. The SVM-polynomial classified $55.1\%$ of the trees as structured and $44.9\%$ as non-structured. In the KNN classification, $84.5\%$ of the trees were classified as structured and the remaining $15.5\%$ classified as non-structured. For trees from the FCs only, the SVM-radial most often classified trees as structured $86.6\%$ and $13.4\%$ as non-structured. The SVM-polynomial mostly classified FCs trees as structured $99.6\%$ and $0.4\%$ as non-structured. In the KNN classification, $80.9\%$ of the trees were classified as structured and $19.1\%$ as non-structured.

### Details of the computing environment

The computing system used to perform the analyses is a 64-bit standalone server running Scientific Linux 7.5 with 64 GB RAM and 2 processors (AMD Opteron (TM) Processor 6274). Each processor had 32 cores with 2 threads per core. Time taken for dataset simulations and real data analysis are shown in Table  3. The time taken in seconds to build classification models under 10-cross-validation is shown in Table 2.

**Table 3:** Execution times for tree dataset simulations and real data analysis

Simulating tree datasets	Non-structured	Structured
Baseline	8 hours, 13 minutes	1 day, 3 hours, 39 minutes
Varied tree size	8 hours, 36 minutes	2 days, 8 hours, 40 minutes
Varied parameters	7 hours, 1 minute	1 day, 10 hours, 17 minutes
Varied tree size & parameters	7 hours, 3 minutes	1 day, 13 hours, 38 minutes
Real data	General population ($n=357$)	Fishing communities and General population ($n=571$)
Calculating tree statistics (250) bootstraps	4 hours, 31 minutes	19 hours, 3 minutes
Calculating tree statistics (1000) bootstraps	18 hours, 23 minutes	3 days, 1 hour, 42 minutes

## Discussion

A system of equations that depicted our model was defined. The obtained expression for $R_0$ using the next generation matrix was used to compute the basic reproductive number under some parameter setting as explained in the methods. This enabled incorporation of $R_0$ in our simulation procedure. The tree classification procedure was also validated by investigating whether classification models were robust to changes in both parameter values and tree size. SVM models were more robust as there were almost no differences observed in the classification performance for the trees. DT and KNN were less robust. This suggests that our tree simulation and classification procedures give promising results.

The two-sample Kolmogorov-Smirnov, Cucconi and Podgor-Gastwirth tests did well in establishing whether the distributions for the tree statistics were different regarding the structure of a population from which the trees were drawn. The tests distinguished the distributions for all the tree statistics for trees from structured and non-structured populations in dataset 1. Only the distributions for the number of cherries and the ladder length for trees from structured and non-structured populations failed to be distinguished using these three tests. The box plots displayed some differences in the tree statistics. The box plots revealed that tree statistics values were slightly higher and more dispersed in the structured population compared to the non-structured population. Further research is needed to establish how many simulated trees and tips in each tree are sufficient to detect significant differences between distributions of computed tree statistics for structured and non-structured populations.

The classification procedure performed very well in distinguishing between trees from structured and non-structured populations. The 10-fold cross validated classification results had mean accuracy of at least 0.78 in all the classifiers that we used. The computed ROC with its corresponding AUC shows that the classifiers’ performance was excellent with AUC values of at least 0.84 for all the classifiers used. From the results, we observe that dataset 1 had the best classification results with a mean accuracy of 0.99 for SVM-radial and SVM-polynomial. This is the case because dataset 1 provided more information for the learning algorithms as we used a constant number of tips of 350 and 200 for non-structured and structured population, respectively for dataset 1, while tips were varied in the interval (300,  400) for dataset 2.

Our study procedure is applicable to real data in terms of informing the choice of phylogenetic tree analysis method (structured or non-structured). Given a real dataset of phylogenetic trees, the study procedure provided insight into the structure of the population under study.This was done by classifying trees as either from a structured or a non structured population using classifiers that had been trained using the simulated trees for dataset 1. We found that tree shape statistics ably provide insight into the population structure underlying transmission patterns of HIV using actual genomic data. Classifiers built on simulated data were able to detect beyond chance the assumed underlying population structure for a combination of general population and fishing communities. In addition, trees from individual populations of GP and FCs were majorly classified as structured rather than non-structured. The fact that the tree statistics can to a certain extent reveal underlying population structure is a proof-of-principle that tree shape statistics are informative.

## Conclusion

We incorporated $R_0$ in our simulation procedure. The classification models were robust to changes in both parameter values and tree size. The structure from which trees were drawn; *that is*, from a structured or a non-structured population was revealed by the classification techniques, the two-sample Kolmogorov-Smirnov, Cucconi and Podgor-Gastwirth tests and the box plots. Other classification procedures using supervised learning algorithms like random decision forest and unsupervised learning algorithms like clustering can be used in further research. The developed study procedure is applicable to real data, in terms of informing the choice of phylogenetic tree analysis methods.

## Methods

### Model design of structured and non-structured host populations

In this study, we considered the dynamics of both structured and non-structured populations. The structured population was broken down into two sub-populations, such that individuals within these sub-populations were indistinguishable, while there was a difference between sub-populations [34]. The choice of two sub-populations was supported by previous work where two groups were used to study population structure. [3, 17, 35].

**Fig. 5:**
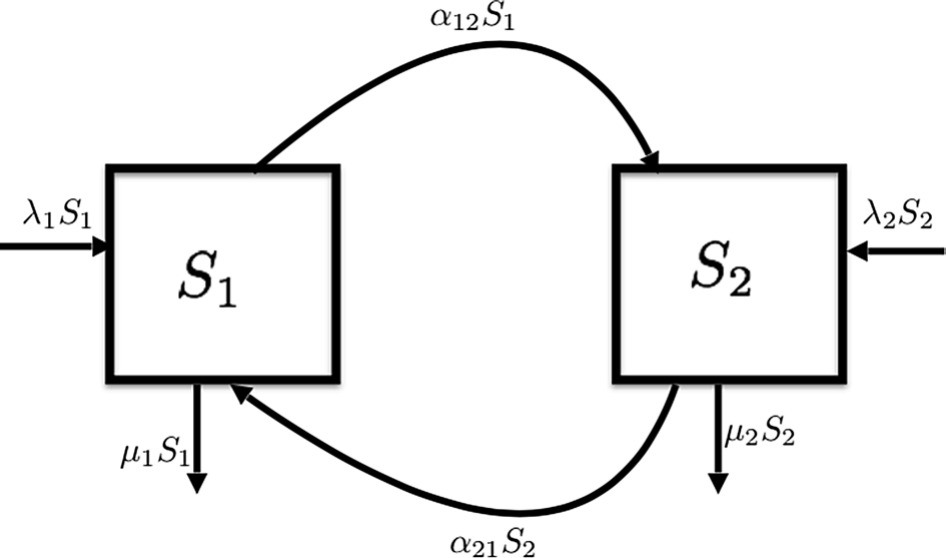
A structured host population with two sub-populations $S_1$ and $S_2$

The structured population consisted of sub-populations $S_1$ and $S_2$, as shown in Fig. 5. Only three main events were allowed to occur in a given sub-population: birth, migration and death. All of these events occurred at different rates between sub-populations. The rate at which an individual in sub-population $S_i$ gave birth to another within that sub-population was $\lambda _i$, for $i \in \{1, 2\}$. An individual in $S_i$ died at a rate of $\mu _i$, for $i \in \{1, 2\}$. An individual from $S_i$ migrated to $S_j$ at a migration rate of $\alpha _{ij}$, where $i, j \in \{1, 2\}$.

Unlike the structured population, all individuals behaved uniformly in the non-structured population. In other words, there were no sub-populations separating a non-structured population. The simulation of a non-structured population followed a birth-death process without migration events.

### Phylogenetic tree simulation in structured and non-structured populations

To simulate phylogenetic trees, we accounted for all of the possible events that can occur during the evolution of a population. For the structured population, we let $N_i(t)$ represent the number of present lineages in $S_i$ at time *t*, for $i \in \{1, 2\}$. A lineage is representative of a single infected individual. We set the waiting time to any event in $S_i$ to follow an exponential distribution with parameter $N_i(t)(\lambda _i + \mu _i + \alpha _{ij})$, where $i, j \in \{1, 2\}$.

Therefore, the density for the waiting time to an event in $S_i$ is given by:

$$\begin{aligned} f(x)= \left\{ \begin{array}{ll} N_i(t) (\lambda _i + \mu _i + \alpha _{ij}) \exp ^{-N_i(t)(\lambda _i + \mu _i + \alpha _{ij})x} &{} x \ge 0 \\ 0 &{} x < 0. \\ \end{array} \right. \end{aligned}$$

Each of our tree simulation process started with one individual in either $S_1$ or $S_2$. Any of the three events (birth, migration or death) then occured randomly.

To determine the sub-population where proceeding events happened, we defined sub-total rates in $S_i$ as $T_{Si}=\lambda _i + \mu _i + \alpha _{ij}$, for $i, j \in \{1, 2\}$. We randomly drew a number between 0 and 1, say *n*.

- If
  $$\begin{aligned} n \le \frac{T_{Si}}{T_{Si} + T_{Sj}}, \end{aligned}$$

the next event happened in $S_i$ and if the condition was not satisfied, then the event happened in $S_j$. Assuming that the selected sub-population for the next event is $S_i$, we determined the next event by randomly drawing a number between 0 and 1, say $\sigma$.

(i) If
  $$\begin{aligned} \sigma \le \frac{\lambda _i}{T_{Si}}, \end{aligned}$$
  then the event was a birth.
(ii) If
  $$\begin{aligned} \frac{\lambda _i }{T_{Si}} < \sigma \le \frac{\lambda _i + \alpha _{ij}}{T_{Si}}, \end{aligned}$$
  then the event was a migration.
(iii) Otherwise, the event was a death.

The simulation was terminated after reaching a given number of extant lineages. However, the simulations can be stopped after a given time *t* or age of the tree. Our simulation procedure was implemented in Python software, version 3.7.3. “ETE 3 Toolkit” was used [36]. We simulated two sets of phylogenetic trees from both structured and non-structured populations.

### Incorporating $R_0$ in phylogenetic tree simulation

The basic reproductive number, denoted as $R_0$, is one of the important disease epidemiological parameters that can be estimated directly from genomic phylogenies (Stadler et al. 2012) [37]. $R_0$ measures the number of secondary cases caused by one primary infection being introduced into an all-susceptible population [38]. In its simplest form, $R_0$ depends on contact rate, probability of a contact producing an infection (susceptible individual getting infected on contact with an infected individual) and the duration of the infectious period [38]. For infectious diseases, a value of $R_0>$ 1 is associated with infection outbreak and persistence [38]. On the other hand, if $R_0 \le 1$, then minor outbreaks with probability of extinction of one, will be realized [38].

We explored the deterministic nature of the birth-death model process, this was aimed at establishing conditions for which the infection (process) persists. This informed choice of parameters for which the simulation process was feasible. The following system of ordinary differential equations describes the dynamics of an infection across the two sub-populations $S_1$ and $S_2$ shown in Fig. 5 with birth, death and migration parameters as previously described.

1 $$\begin{aligned} \dfrac{dS_1}{dt}&= (\lambda _1S_1 + \alpha _{21}S_2) - (\mu _1S_1 + \alpha _{12}S_1), \end{aligned}$$

2 $$\begin{aligned} \dfrac{dS_2}{dt}&= (\lambda _2S_2 + \alpha _{12}S_1) - (\mu _2S_2 + \alpha _{21}S_2). \end{aligned}$$

We obtained equilibrium points by setting the above equations to zero,

$$\begin{aligned} \dfrac{dS_1}{dt}&= 0 , \\ \dfrac{dS_2}{dt}&= 0 \end{aligned}$$

The Disease Free Equilibrium (DFE) of the system is given by $(S_1^*, S_2^*) = (0,0)$ .

We then computed $R_0$ of this system using the next generation matrix method [39, 40]. Unlike typical epidemiological models, both compartments are representative of infectives. We let *X* be defined as:

$$\begin{aligned} X&= \begin{bmatrix} S_1 \\ S_2 \end{bmatrix} \end{aligned}$$

We then constructed matrices ${\mathcal {F}}$ and ${\mathcal {V}}$ for new and other infection terms in the respective compartments as:

$$\begin{aligned} {\mathcal {F}}&= \begin{bmatrix} \lambda _1S_1 \\ \lambda _2S_2 \end{bmatrix} = \begin{bmatrix} f_1 \\ f_2 \end{bmatrix} , ~~~~ {\mathcal {V}} = \begin{bmatrix} \mu _1S_1 + \alpha _{12}S_1 - \alpha _{21}S_2 \\ \mu _2S_2 + \alpha _{21}S_2 - \alpha _{12}S_1 \end{bmatrix} = \begin{bmatrix} v_1 \\ v_2 \end{bmatrix} \end{aligned}$$

Jacobian matrices of ${\mathcal {F}}$ and ${\mathcal {V}}$ at the DFE were obtained as:

$$\begin{aligned} F&= \begin{bmatrix} \dfrac{\partial f_1}{\partial S_1} &{} \dfrac{\partial f_1}{\partial S_2} \\ \\ \dfrac{\partial f_2}{\partial S_1} &{} \dfrac{\partial f_2}{\partial S_2} \end{bmatrix} _{(0,0)} = \begin{bmatrix} \lambda _1 &{} 0 \\ 0 &{} \lambda 2 \end{bmatrix}. \end{aligned}$$

Similarly,

$$\begin{aligned} V&= \begin{bmatrix} \dfrac{\partial v_1}{\partial S_1} &{} \dfrac{\partial v_1}{\partial S_2} \\ \\ \dfrac{\partial v_2}{\partial S_1} &{} \dfrac{\partial v_2}{\partial S_2} \end{bmatrix} _{(0,0)} = \begin{bmatrix} \alpha _{12} + \mu _1 &{} -\alpha _{21} \\ -\alpha _{12} &{} \alpha _{21}+\mu _2 \end{bmatrix}. \end{aligned}$$

The inverse of *V* was computed and it is given as:

$$\begin{aligned} V^{-1}&= \begin{bmatrix} \frac{1}{\alpha _{12} + \mu _{1}} - \frac{\alpha _{12} \alpha _{21}}{{\left( \alpha _{12}+ \mu _{1}\right) }^{2} {\left( \frac{\alpha _{12} \alpha _{21}}{\alpha _{12} + \mu _{1}} - \alpha _{21} - \mu _{2}\right) }} &{} -\frac{\alpha _{21}}{{\left( \alpha _{12} + \mu _{1}\right) } {\left( \frac{\alpha _{12} \alpha _{21}}{\alpha _{12} + \mu _{1}} - \alpha _{21} - \mu _{2}\right) }} \\ -\frac{\alpha _{12}}{{\left( \alpha _{12} + \mu _{1}\right) } {\left( \frac{\alpha _{12} \alpha _{21}}{\alpha _{12} + \mu _{1}} - \alpha _{21} - \mu _{2}\right) }} &{} -\frac{1}{\frac{\alpha _{12} \alpha _{21}}{\alpha _{12} + \mu _{1}} - \alpha _{21} - \mu _{2}} \end{bmatrix} , \end{aligned}$$

We computed the next generation matrix given as $FV^{-1}$ and obtained corresponding eigenvalues. $R_0$ is the maximum eigenvalue. For the system of equations defining our model, $R_0$ was given as:

$$\begin{aligned} R_0&= \dfrac{\alpha _{21} \lambda _{1} + \alpha _{12} \lambda _{2} + \lambda _{2} \mu _{1} + \lambda _{1} \mu _{2} + \sqrt{\Delta }}{2 \, {\left( \alpha _{21} \mu _{1} + {\left( \alpha _{12} + \mu _{1}\right) } \mu _{2}\right) }} \end{aligned}$$

Where,

$$\begin{aligned} \begin{aligned} \Delta&= \alpha _{21}^{2} \lambda _{1}^{2} + 2 \, \alpha _{12} \alpha _{21} \lambda _{1} \lambda _{2} + \alpha _{12}^{2} \lambda _{2}^{2} + \lambda _{2}^{2} \mu _{1}^{2} + \lambda _{1}^{2} \mu _{2}^{2} - 2 \, {\left( \alpha _{21} \lambda _{1} \lambda _{2} - \alpha _{12} \lambda _{2}^{2}\right) } \mu _{1} \\&\quad + 2 \, {\left( \alpha _{21} \lambda _{1}^{2} - \alpha _{12} \lambda _{1} \lambda _{2} - \lambda _{1} \lambda _{2} \mu _{1}\right) } \mu _{2} \end{aligned} \end{aligned}$$

The above expression of $R_0$ applies to the general case of a structured population with two sub-populations, $S_1$ and $S_2$. We explored two cases for obtaining the $R_0$, in the first case, we considered a single population with no sub-populations. We had only birth and death with no migration rate parameters. This was the scenario under a non-structured population. In the second case, birth, death and migration rate parameters in one sub-population were multiplied by a factor *k* of the parameters in other sub-population. This case represented a structured population. In both cases, we established the relationship among parameters for which $R_0>1$.

1. $$\lambda _1=\lambda _2=\lambda$$, $$\mu _1=\mu _2=\mu$$, $$\alpha _{12}=\alpha _{21}=0$$,
  $$R_0 = \dfrac{\lambda }{\mu } ~~~\text {for}~ R_0> 1 \implies \lambda > \mu$$.
2. $$\lambda _2=k\lambda _1$$, $$\mu _2=k\mu _1$$, $$\alpha _{21}=k\alpha _{12}$$, where $$k>0$$,
  $$R_0 = \dfrac{\lambda _1}{2\alpha _{12} + \mu _1}~~\text {for}~~R_0> 1 \implies \lambda _1 > 2\alpha _{12} + \mu _1$$.

### Choice of parameters used in the phylogenetic tree simulations

Since our interest was to apply our methods to real dataset like for HIV/AIDS sequence data, the parameters we used in the phylogenetic tree simulations were based on literature related to HIV/AIDS in Uganda. For the death rate, [41] reported that the estimated deaths of adults due to HIV/AIDS was 21,000 (17,000  29,000) out of a total of 1,500,000 (1,400,000  1,600,000) adults living with HIV/AIDS. This translates into death rate of 0.014 (0.01  0.02). Since [42] observed that HIV prevalence was three times higher in communities at high risk of getting infected compared to the general population, we multiplied by 3 to obtain the death rate parameter for the high risk sub-population in a structured population. For $R_0$, the choice was based on the work of [43] where we used $R_0$ of 4.99 (0.45, 6.34) for non-structured and 9.09 (4.18, 36.75) for a structured population. For the migration rate parameter, the value used was 0.3 (0.18, 0.44) for low risk sub-population and 0.2 (0.10, 0.33) for high risk sub-population in a structured population. This was based on the work of [44]. We then computed birth rate parameter based on the formula for $R_0$. Parameter values from literature which are relevant to our study are shown in Table 4.

**Table 4:** Parameter values used for simulating phylogenetic trees for structured and non-structured populations and their corresponding parameter values from literature

Structured population	Sub-population 1	Sub-population 2
Basic reproductive number ($R_{0}$)	4.99	9.09
Birth rate ($\lambda$)	3.0639	4.0178
Death rate ($\mu$)	0.014	0.042
Migration rate ($\alpha$)	0.3	0.2
Number of tips (*n*)	350	200
Number of trees	250	250
Non-structured population		
$R_0$	4.99	
$\lambda$	0.0699	
$\mu$	0.014	
*n*	350	
Number of trees	500	

From literature	Values	References
HIV/AIDS related deaths ($\mu$)	0.014(0.01, 0.02)	[41, 42]
$R_0$	4.99(0.45, 6.34) for low risk sub-population & 9.09(4.18, 36.75) for high sub-population	[43]
Out-migration ($\alpha$)	0.30(0.18, 0.44) for low risk sub-population & 0.20(0.10, 0.33) for high risk sub-population	[44]

### Sets of simulated trees

For dataset 1 (baseline dataset), the parameters for a non-structured population were: $\mu =0.014, R_0=4.99, \lambda =\mu (R_0)$, number of tips was 350 and number of trees was 500. For the structured population, in sub-population 1 (low risk), the parameters used were: $\mu _1=0.014,~\alpha _{12}=0.3,~R_{01}=4.99,~\lambda _1=R_{01}(2\alpha _{12}+\mu _1)$, the number of tips was 350 and the number of trees was 250. For sub-population 2 (high risk), the parameters used were: $\mu _2=3(0.014),~\alpha _{21}=0.2,~R_{02}=9.09,~\lambda _2=R_{02}(2\alpha _{21}+\mu _2)$, the number of tips was 200 and the number of trees was 250.

For dataset 2, the number of tips was varied while keeping other parameter values for dataset 1 constant. For the structured population, the number of tips was varied in the interval (300,  400) for sub-population 1 and (150,  250) for sub-population 2. For a non-structured population, tips were varied in the interval (300,  400). A summary of the parameter values used for dataset 1 is shown in Table 4.

### Estimating the tree statistics from the simulated trees

The shapes of trees simulated from both structured and non-structured populations were examined using estimates of tree statistics. These tree statistics included: number of cherries; Sackin, Colless and total cophenetic indices; ladder length; maximum width; maximum depth and width to depth ratio index. A detailed description on the number of cherries was done by [18, 29, 32]. For the Sackin index, details can be found in the work of [18, 24]. The Colless index was defined and described by [24, 45]. The total cophenetic index is one of the new indices for phylogenetic trees, which is defined in the work of [33]. Definitions and descriptions for ladder length, maximum depth of a tree, maximum width of a tree, and width to depth ratio index were done by [25]. A summary of the definitions of the eight tree statistics is shown in Table 5. Fig. 1 shows a simulated tree with 5 tips and Table 6 shows how the corresponding tree statistics were computed. All computations for the eight statistics were implemented in Python software, version 3.7.3.

**Table 5:** Definitions for the tree statistics

Tree statistics	Definition	References
Cherry	Pair of leaves that is adjacent to a common ancestor node.	[18, 29, 32]
Normalized number of cherries	Number of cherries divided by half the number of tips in a tree.	[46]
Sackin index	Sum of all the number of edges from a leaf to a root for each of a leaf in a tree.	[18, 24]
Normalized Sackin index	Sackin index divided by $(0.5\times (n(n+1))-1)$, where *n* is the number of tips.	[46]
Colless index	Sum of absolute differences between left and right hand leaves (terminal tips) subtended at each internal node of a tree, the root inclusive.	[24, 45]
Normalized Colless index	Colless index divided by $\frac{(n-1)(n-2)}{2}$, where *n* is the number of tips.	[45]
Total cophenetic index	Sum of all depths of the lowest common ancestor for all pairs of leaves in a tree.	[33]
Ladder length	Ratio of maximum number of connected internal nodes with a single descendant leaf to number of leaves in a tree.	[25]
Maximum depth	Maximum number of edges from a leaf to a root for all the leaves in a tree.	[25]
Maximum width	Maximum number of nodes for each possible depth of a tree.	[25]
Width-depth ratio	Ratio of maximum width of a tree to its maximum depth.	[25]

**Table 6:** Computed tree statistics from the illustrated tree in Fig. 1

Tree statistics	Computed value
Number of Cherries	Cherries are formed by tips 1 & 2 and 4 & 5. So the number of cherries is 2.
Standardized number of Cherries	From the formula, $\frac{2}{0.5*5}=0.8$
Sackin index	We consider each leaf and we count the edges to the root, e.g for leaf 1, there are 3 edges to the root. The value of the Sackin index becomes 14.
Standardized Sackin index	From the formula, $\frac{14}{0.5 \times 5 \times 6 - 1}=\frac{14}{14}=1$
Colless index	We consider each internal node, e.g for internal node C, the difference between left and right tips subtended is 1. Adding such values for each internal node results in 2 as the Colless index.
Standardized Colless index	From the formula, $\frac{2}{4\times 3|2}=0.3333$
Total cophenetic index	$(1,2),(1,3),(1,4),(1,5),(2,3),(2,4),(2,5),(3,4),(3,5) \& (4,5)$ are the possible pairs. The corresponding cophenetic values are $2,0,0,0,0,0,0,1,1 \& 2$, respectively. Sum of all possible pairs is 6.
Ladder length	One internal node C has a single child descendant leaf, ladder length therefore is $\frac{1}{5}=0.2.$
Maximum depth	Depth for tips 1,2,3,4 & 5 are 3,3,2,3 & 3. Since 3 is the highest, it is the maximum depth.
Maximum width	Depth for tips 1,2,3,4 & 5 are 3,3,2,3 & 3 respectively. Depth for internal nodes A,B,C,D & E are 0,1,1,2 & 2. Depth 3 has the highest number of nodes and it is 4. Maximum width becomes 4.
Width-depth ratio	$\frac{4}{3}=1.3333$

### Comparing tree statistics estimated from structured and non-structured populations

We investigated whether the estimated tree statistics could be used to distinguish between structured and non-structured host populations. The tree statistics were visualised using box plots for both populations. These were helpful for summarizing location and dispersion of the tree statistics.

Because the box plots could not give concise distinction in the tree statistics between populations, we first compared the distributions of the tree statistics for both populations using a two-sample Kolmogorov-Smirnov test [47]. For a given tree statistic, we investigated whether this test distinguished the corresponding distributions under both populations. From the computed *p*-values, Kolmogorov-Smirnov test distinguished between trees from either structured or non-structured population at a level of 0.05. For this test, the *D* parameter is the Kolmogorov-Smirnov statistic, which measures the distance between the two distributions under comparison. The larger the *D* parameter, the smaller the *p*-value, and the more distant (different) the two distributions under comparison are. In addition, we used two non-parametric tests, which included Cucconi and Podgor-Gastwirth tests. These are also two-sample tests which detect whether the two underlying samples are distinct using both location and scale parameters. These two and some others were described by [48]. Box plots, two-sample Kolmogorov-Smirnov, Cucconi and Podgor-Gastwirth tests were implemented in R, version 4.0.2. R package ‘ggplot2’ [49] was used for box plots drawing.

### Classification of simulated trees as either from a structured or a non-structured population based on their tree statistics

We used various classification algorithms to determine the type of population (structured or non-structured) from which a given set of trees were simulated based on their estimated tree statistics. The classification algorithms that we used were; K-nearest neighbour (KNN) [50], support vector machine (SVM) [51, 52] and decision trees (DT) [53]. Table 7 gives descriptions of these classifiers.

**Table 7:** Descriptions for machine learning techniques

Machine learning technique	Description	References
K-nearest neighbour (KNN)	KNN classifies an object based on closest training examples in the feature space. KNN is a supervised machine learning technique where data is divided into two sets: a training and a test set. The training set is used to train the machine (learning), while the test set is used to determine the classes of the given objects (actual classification). Given an unknown sample $(k_0)$ to be classified and a training data set, the distances between $k_0$ and all samples in the training set are computed. The number of neighbours (*k*) that have the shortest distance (closest) to $k_0$ are identified. And $k_0$ will be inferred to belong to the class where its *k* closest neighbours come from. Some of the distance metrics that can be used in the KNN classification include: Eucledian, Eucledian squared, City-block and Chebychev.	[50]
Support vector machine (SVM)	SVM is both a supervised learning and a binary classification method. It finds the best separating hyperplane between two classes of the training samples in the feature space. Suppose we have *n* sample points in the training set, where each sample point $\mathbf{x }_i$, has *k* attributes and each belongs to one of two classes. Let us denote the classes as either 1 or $-1$, the sample points are denoted as $(\mathbf{x }_i,y_i)$, where $i=1,...,n$, $y_i \in \{ -1,1\}$ and $\mathbf{x } \in {\mathbb {R}}^k$. For the case when the data are separable and $k=2$, a line separating between the two classes is easily drawn. In circumstances where $k>2$ and the data are still separable, a hyperplane separates the two classes. For a case when the data are not linearly separable, the data are transformed using kernel functions. Some of the commonly used functions include radial basis kernel, linear kernel, polynomial kernel and the sigmoidal kernel.	[51, 52]
Decision tree (DT)	DT procedure divides a data set into subdivisions basing on a set of tests that are defined at each branch or a node. From the given data, a tree is constructed which is composed of a root, internal nodes which are known as splits and a set of leaves. The leaves are the terminal nodes. Data are classified according to the decision framework defined by the tree. It is the leaf nodes that are assigned the label class. The assignment is done according to the leaf node into which the observation falls. The learning algorithms define splits at each internal node of a decision tree from the training data set. For an accurate decision tree, the training data should be of high quality so that the relations between the features and classes can be easily learned.	[53]

Here, we aimed at classifying the simulated trees into two main classes; *that is*, a structured and a non-structured population. To establish a proportion to be used for training, we tried out different proportions of data; *that is*, $30\%$, $50\%$ and $70\%$ for training and with the corresponding remaining proportions for testing of the classifiers. We then performed grid search for the parameters to obtain optimal models to be used for classification. The classification procedure for the optimal models for KNN, SVM and DT was implemented in R software, version 4.0.2. R package ‘e107’ [54] was used in the classification.

### Evaluation and cross-validation of tree classifiers

We compared the performance of all the three classification techniques using measures such as sensitivity, specificity and accuracy rate [55]. We also evaluated the performance of the classifiers using receiver operating characteristics (ROC) curves. For ROC curve, true positive rate (sensitivity) is plotted against false positive rate (1-specificity). The area under the curve (AUC) for the ROC curves was computed. Some analysis and comparison of ROC curves are given in [56]. In our study, the AUC quantifies the overall ability of the classifier to discriminate between trees simulated from structured and non-structured populations. The AUC ranges between 0.5 and 1 for a realistic classifier, as reported by [57]. A perfect classifier (one that has zero false positives and zero false negatives) has an area of 1. The closer the value of AUC to 1, the better the classifier performance.

We performed 10-fold cross validation. Under this, a given dataset is divided into 10 equal portions and each of the portions is used as a testing set, while the remaining 9 are used for model training. We then obtained the means for the measures that we used for evaluation of classifiers. Cross validation gives realistic evaluation of model performance as done in some studies [58, 59]. Parameter tuning of model parameters and cross validation was done with help of ‘caret’ R package [60]. The evaluation and cross-validation of tree classifiers was implemented in R software, version 4.0.2.

### Sensitivity analysis

To determine whether the classifiers were robust to different choices of model parameters and to size of the trees, we simulated three sets of 250 structured and 250 non-structured trees (500 in total) with (i) randomly selected parameters, (ii) a random tree size and (iii) random parameters and random tree size. Parameters and tree sizes were obtained using Latin hyper cube sampling as implemented in the “SMT” python “toolkit”. Parameters were selected such that $R_0$ was in the intervals (0.45,  6.34) and (4.18,  36.75) for sub-population 1 and 2, respectively. Similarly, tree size was varied to lie in the intervals (300,  400) and (250, 300) for sub population 1 and 2, respectively. Selected parameters and tree sizes were similar to the real-world populations that were later investigated. Simulated trees were then classified under 10-fold cross-validation.

### Application of the classification procedure to real-world data

To evaluate the classifiers’ performance on real-world data within known epidemiology, we used the classifiers on sequence data from two key populations in Uganda whose underlying HIV-1 transmission dynamics have been previously described by [61]. We applied our classifiers on phylogenetic trees from previously published HIV$-1$ sequence data from the general population (GP) and fishing communities (FCs) of Uganda, Bbosa et al. [61]. The sequences were retrieved from the NCBI nucleotide database, accession numbers MG434786 to MG435347. The data comprised of 357 sequences from the GP and 221 sequences from FCs. Two sets of trees were generated; (i) only GP (ii) only FCs and (iii) the combination of GP and FCs. Sequences were aligned using “clustalw” [62], ahead of generating 1000 bootstraps of maximum likelihood trees using IQ-TREE of Nguyen et al. [63] with UFBoot2 of Hoang et al. [64]. SVM, DT and KNN classifiers trained at 10-fold cross validation at baseline were used to predict the population structure of the bootstraps. We classified maximum likelihood 1000 tree bootstraps generated from three sets of data including; sequences from GP, FCs and both GP and FCs.

## Data Availability

Raw data and codes used during the current study are available at https://github.com/HassanKayondo/TreeShape

## Abbreviations

AUC: Area under the curve
DFE: Disease Free Equilibrium
DT: Decision tree
FCs: Fishing communities
GP: General population
HIV: Human Immunodeficiency Virus
KNN: K-nearest neighbor
$R_0$: Basic reproductive number
SVM: Support vector machine
ROC: Receiver operating characteristic
